# A Case of Cutaneous Plasmablastic Lymphoma in HIV/AIDS with Disseminated *Cryptococcus*

**DOI:** 10.1155/2013/862585

**Published:** 2013-11-26

**Authors:** Jun Gong, Serhan Alkan, Sidharth Anand

**Affiliations:** ^1^Department of Internal Medicine, Cedars-Sinai Medical Center, 8700 Beverly Boulevard, No. 5512, Los Angeles, CA 90048, USA; ^2^Department of Pathology and Laboratory Medicine, Cytogenetics, Cedars-Sinai Medical Center, 8700 Beverly Boulevard, South Tower, Room 4707, Los Angeles, CA 90048, USA

## Abstract

We present a case of a patient with HIV/AIDS who presented with a tender left lower extremity cutaneous mass over a site of previous cryptococcal infection and was found to have plasmablastic lymphoma (PBL). The incidence of PBL is estimated to account for less than 5% of all cases of non-Hodgkin lymphoma (NHL) in HIV-positive individuals. In fact, there were only two reports of extraoral PBL at the time of a 2003 review. PBL in HIV-positive individuals is an aggressive malignancy that tends to occur in middle-aged males with low CD4 counts, high viral loads, and chronic HIV infection. The definitive diagnosis can be made with biopsy which typically shows malignant lymphoid cells that stain positive for plasma cell markers and negative for B-cell markers. The most common treatment is chemotherapy with cyclophosphamide, doxorubicin, vincristine, and prednisone (CHOP) or CHOP-like regimens, but the overall survival rate is poor despite its relative responsiveness to chemotherapy. This case highlights the challenges that remain in improving clinical outcomes, the importance of antiretroviral therapy and HIV disease control, and a potential association between a chronic inflammatory state caused by disseminated *Cryptococcus* and tumorigenesis in individuals with PBL.

## 1. Introduction

Kaposi's sarcoma, non-Hodgkin lymphoma (NHL), and invasive cervical carcinoma are among the three AIDS-defining malignancies, and, of these, Kaposi's sarcoma and AIDS-related non-Hodgkin lymphoma are the most common [1]. AIDS-related NHL can be categorized into systemic NHL, primary CNS lymphoma, and primary effusion lymphoma [2]. In HIV-infected individuals, systemic NHL is the most common and can be further divided into subtypes such as diffuse large B-cell lymphoma (DLBCL), Burkitt's lymphoma, T-cell lymphoma, and plasmablastic lymphoma (PBL), to name a few [2]. Others have described plasmablastic lymphoma as a unique variant of DLBCL with a propensity to develop in HIV-positive patients with frequent involvement of the oral cavity [3–5]. At the time of a 2003 review, there were only 2 reports of extraoral plasmablastic lymphoma [4]. Of the NHL subtypes seen in HIV-positive individuals, the incidence of DLBCL is estimated to account for almost 50% of the cases while the incidence of plasmablastic lymphoma is estimated to account for less than 5% of the cases [3, 4]. The median age of presentation for PBL is 38 years with a greater predominance in males [5]. PBL also tends to occur in HIV-positive individuals with absolute CD4 counts less than 200 CMM, mean viral loads greater than 80,000 copies/mL, and an average duration of HIV infection of 5 years [5]. We present a rare case of a 63-year-old HIV-positive male who was found to have plasmablastic lymphoma on biopsy of a tender left lower extremity cutaneous mass occurring over a previous site of cryptococcal infection.

## 2. Case Report

**Figure 1:**
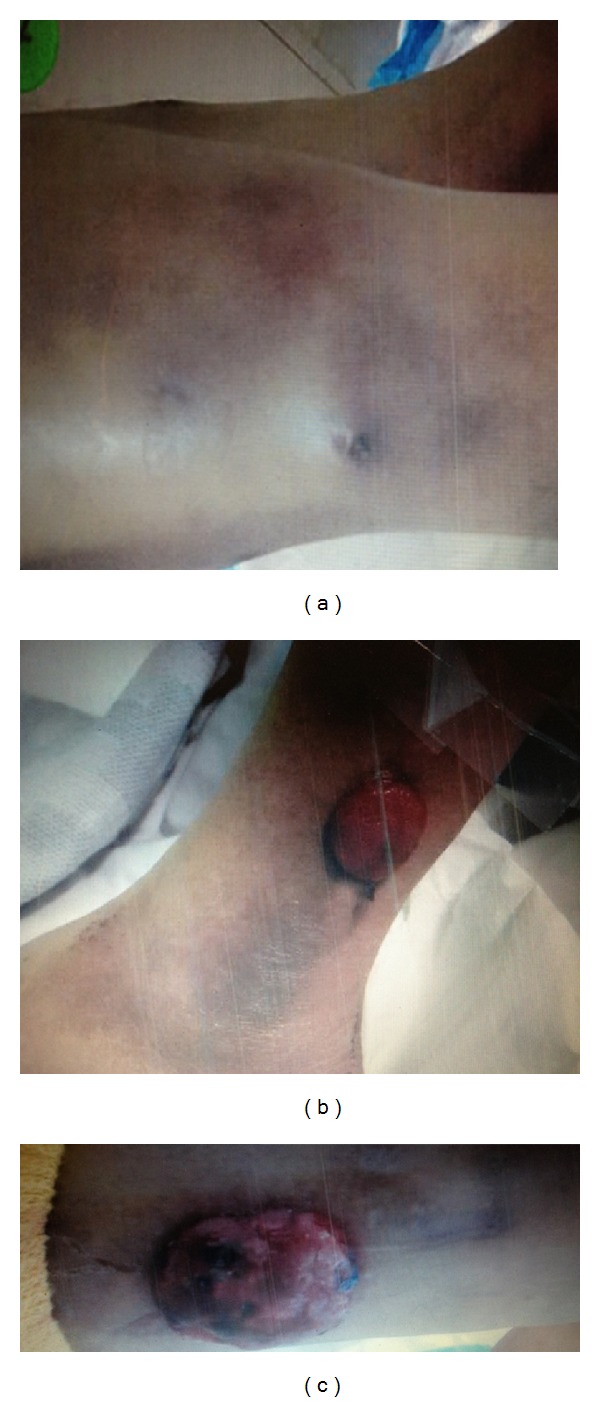
Physical exam of the right thigh revealed two fluctuant, well-circumscribed, circular lesions approximately 3 cm in diameter that were tender to palpation and non-mobile (a). Examination of the left lower extremity showed a fleshy-appearing, pinkish, ovular exophytic mass approximately 5 cm in diameter that was tender to palpation (b) and (c).

A 63-year-old male with a history of HIV/AIDS (last known CD4 count of 279 CMM) and disseminated *Cryptococcus* presented to the emergency department with a painful protuberant left lower extremity cutaneous mass. He initially experienced symptoms of bilateral lower extremity lesions four months prior to presentation, during which surgical biopsy of a left lateral calf mass yielded necrotizing granulomatous inflammation with *Cryptococcus*. During that admission, he was treated with amphotericin and flucytosine and discharged home on oral fluconazole. These cutaneous lesions persisted and he was treated with periodic incision and drainage of these areas; however, this new lesion on his left lower extremity appeared over several weeks prior to this admission and brought him to the emergency room. His physical exam was remarkable for the following skin findings: (1) the anterolateral aspect of his right thigh had two fluctuant, well-circumscribed, circular lesions approximately 3 cm in diameter that were tender to palpation and nonmobile and (2) his left lower extremity had a fleshy-appearing, pinkish, ovular exophytic mass approximately 5 cm in diameter that was tender to palpation and appeared to be growing over an area of previous *Cryptococcus* infection (Figure 1).

**Figure 2:**
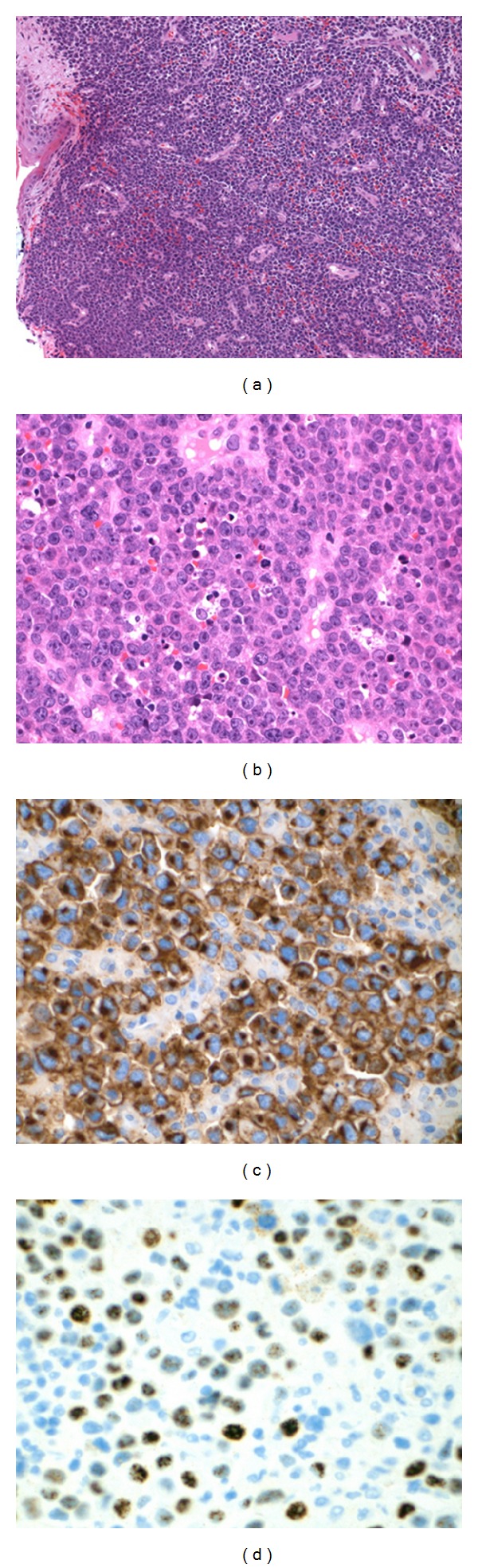
Cutaneous biopsy revealed a diffuse sheet of subepidermal large lymphoma cells with high mitotic rate and occasional tingible body macrophages (a) and (b). Immunohistochemical staining showed expression of CD138 (c) and EBV in situ hybridization (EBER transcript) revealed many of the lymphoma cells infected by EBV (d).

**Figure 3:**
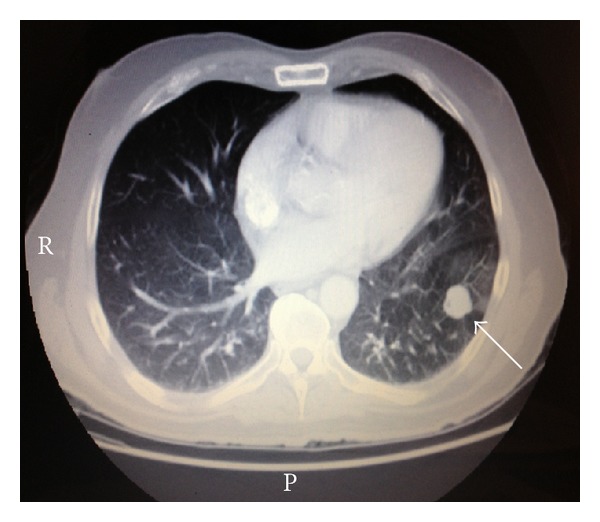
Staging CT scan showed a pulmonary nodule identified in the left lower lobe which is 1.8 × 1.8 cm in diameter.

**Figure 4:**
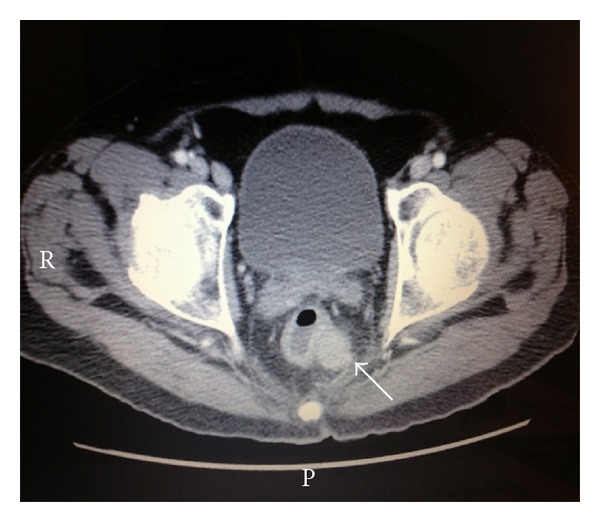
Staging CT scan showed a left perirectal soft tissue mass which is 2.0 × 2.2 cm in diameter.

His laboratories showed a repeat absolute CD4 count of 93 CMM, HIV RNA PCR of 117 copies/mL, and CBC significant for a normocytic, normochromic anemia (with an otherwise unremarkable differential). Given the different appearance of his new left lower extremity lesion, a repeat biopsy was performed. The biopsy of the cutaneous lesion revealed sheets of large cells with an abundant amount of cytoplasm with plasmacytoid features and increased mitosis (Figure 2). In some areas, there was an increased number of tingible body macrophages noted. Immunohistochemical analysis identified malignant lymphoid cells that expressed CD138, CD79a, OCT-2, BOB-1, and MUM-1 with high mitotic rate as noted by Ki67, while the lymphoma cells were negative for CD20, CD30, and HHV-8. A cMYC rearrangement was detected by FISH. Flow cytometric analysis also supported the virtual absence of B-cell markers in the tumor cells. These findings were consistent with plasmablastic lymphoma. A bone marrow biopsy was performed which was negative for any bone marrow involvement, and a staging CT scan showed a pulmonary nodule identified in the left lower lobe which is 1.8 × 1.8 cm in diameter (Figure 3), a left perirectal soft tissue mass 2.0 × 2.2 cm in diameter (Figure 4), and a focal area of left anal wall thickening 2.4 × 1.6 cm in diameter. Further biopsies were not pursued, and the patient was subsequently started on his first cycle of cyclophosphamide, doxorubicin, vincristine, and prednisone (CHOP). The patient was recovering from his first cycle and awaiting his second cycle of CHOP at the time of this report.

## 3. Discussion

 Cutaneous plasmablastic lymphoma is a unique presentation of an already very rare and aggressive subtype of non-Hodgkin lymphoma in HIV-positive individuals. The clinical presentation of plasmablastic lymphoma, being a subtype of NHL, can be characterized by the presence of adenopathy in virtually any location, B symptoms such as fever, night sweats, weight loss, or anorexia, and extranodal involvement with symptoms due to mass effect of extranodal disease [6, 7]. However, the primary site of presentation of plasmablastic lymphoma can vary tremendously. The first cases of reported PBL occurred as oral lesions in HIV-positive patients in the mid 90s [4]. A review of 112 cases of PBL reported that the most common primary lymphoma site was the oral cavity followed by extranodal, nonoral sites such as the nasal and paranasal cavities, skin, anal canal, soft tissues, bone and bone marrow, CNS, mediastinum, and gonads [5]. The next most common site was the nonoral and nonanal components of the GI tract, and the least common were the lymph nodes [5]. A majority of the cases of PBL present as advanced disease (51% of cases with Ann Arbor stage III or IV) while 49% of cases present with stage I or II disease [7]. 

Like other NHL subtypes, the definitive diagnosis of plasmablastic lymphoma is made with biopsy of the involved site. The histopathologic features of PBL include the presence of a high grade lymphoma comprised of plasmablasts that stain positive for plasma cell markers such as CD38, VS38c, MUM1, and CD138 [5]. PBL is also associated with EBV-positivity, the presence of c-MYC rearrangement, and high Ki67 expression [5, 7, 8]. PBL is also characterized by frequent absence of B-cell markers such as CD20 and absence of HHV8 positivity [5]. 

With respect to pathogenesis, PBL has been predominately associated with HIV infection, particularly in individuals with low absolute CD4 counts, high viral loads, and chronic HIV infection [5]. However, cases of PBL in the absence of HIV infection have also been described in association with solid organ transplantation and chronic immunosuppression and autoimmune disease such as Crohn's disease [9]. In addition, latent Epstein-Barr virus (EBV) infection is detected in a majority (70%) of PBL cases [9]. The association between PBL and cryptococcal infection, however, is poorly studied and limited to a few case reports [10, 11].

Given its aggressive nature, a majority of patients with PBL are treated with chemotherapy with the most common chemotherapeutic regimen being cyclophosphamide, doxorubicin, vincristine, and prednisone (CHOP) [7]. One review reports that 23% of cases were treated with more intensive regimens such as HyperCVAD, CODOX-M/IVAC, and EPOCH, 19% were treated with radiotherapy, 10% were treated with intrathecal methotrexate, and 27% were treated with other therapies [7]. R-CHOP appears to be of benefit in a small subset of HIV-positive individuals with CD20-positivity, absolute CD4 counts >100 CMM, or positive MYC rearrangements [7]. There is no reported difference in overall survival between CHOP and more intensive regimens [7]. The overall response rate to chemotherapy is relatively high at 77% with 46% of cases experiencing a complete response, 31% with a partial response, and 23% with no response [7]. However, despite its responsiveness to chemotherapy, plasmablastic lymphoma carries a poor prognosis with a 5-year overall survival rate of 31% and a median overall survival period of 14 months, with most cases having died due to progression of disease [7]. Positive prognostic indicators include early stage of disease, complete response to chemotherapy, lack of bone marrow involvement, and use of antiretroviral therapy while presence of MYC rearrangement is a negative prognostic indicator [7].
